# A new *Chlerogelloides* from northeastern Brazil and French Guiana, with a key to the species (Hymenoptera, Halictidae)

**DOI:** 10.3897/zookeys.185.2551

**Published:** 2012-04-23

**Authors:** Favízia Freitas de Oliveira, Michael S. Engel, Thiago Mahlmann

**Affiliations:** 1 Laboratório de Bionomia, Biogeografia e Sistemática de Insetos (BIOSIS), Departamento de Zoologia, Instituto de Biologia, Universidade Federal da Bahia, Rua Barão de Jeremoabo, s/n, Campus Universitário de Ondina, Salvador, CEP 40170-115, Bahia, Brazil; 2 Division of Entomology, Natural History Museum, and Department of Ecology & Evolutionary Biology, 1501 Crestline Drive – Suite 140, University of Kansas, Lawrence, Kansas 66045, USA

**Keywords:** Apoidea, Anthophila, Halictidae, Halictinae, Augochlorini, *Chlerogelloides*, taxonomy, Brazil

## Abstract

A third species of the rare augochlorine bee genus *Chlerogelloides* Engel et al. (Halictinae, Augochlorini) is described and figured. *Chlerogelloides nexosa* **sp. n.** is most similar to the type species, *Chlerogelloides femoralis* Engel et al., in that both have modified midlegs in the males. The former, however, can be distinguished on the basis of its terminalia, which in some respects more closely resembles those of *Chlerogelloides simplex* Engel and Brooks. Brief comments on the secondary features of males and relationships of the genus are provided. A key to the species of the genus is provided and floral records of *Cordia nodosa* Lam. (Boraginaceae) and *Gonzalagunia dicocca* Cham. & Schltdl. (Rubiaceae) are noted.

## Introduction

Numerous lineages of bees within the New World tribe Augochlorini (Halictinae) are known to have characteristically elongate heads, typically involving an elongation of the malar space and clypeus, and often the supraclypeal area, and presumably adaptations for obtaining nectar from flowers with deep corollas, although few floral records are available (e.g., Engel 2000, 2007, 2009, 2010a, 2010b; Engel and Brooks 2002; Engel and Gonzalez 2009). Among such bees, one of the more peculiar are the species of the genus *Chlerogelloides* Engel et al. (Engel et al. 1997; Engel and Brooks 1999), so named for its superficial resemblance to the more diverse and widespread genus *Chlerogella* Michener (Engel 2009, 2010a), and putatively related to *Chlerogella* and *Ischnomelissa* Engel (Engel 2000). Unlike nearly all of the aforementioned cases of augochlorines with noticeably elongate head, in *Chlerogelloides* the malar space is unmodified and short, but the clypeus remains distinctively elongate. A complete account of the genus has been provided by Engel and Brooks (1999) and Engel (2000). To hitherto known species of *Chlerogelloides* have been reported from Brazil (Amazonas), Colombia (Putumayo), Ecuador (Morona Santiago, Napo, Sucumbíos), French Guiana (Roura), and Peru(Loreto) for *Chlerogelloides femoralis* Engel et al. and Brazil (Amapá) and French Guiana for *Chlerogelloides simplex* Engel and Brooks (Engel et al. 1997; Engel and Brooks 1999; Moure 2007). As the specific epithet suggests, the type species, *Chlerogelloides femoralis*, is also noteworthy among the Augochlorini for its peculiarly modified midlegs in males, the function of which remains unknown but are presumably used in mating. Tragically, no biological information of any kind has been reported for any species of the genus.

Herein we report the discovery of a third species in the genus, specimens of which were captured while visiting flowers of *Cordia nodosa* Lam. (Boraginaceae: sometimes placed in its own order, Boraginales) and *Gonzalagunia dicocca* Cham. & Schltdl. (Gentianales: Rubiaceae), and representing the first occurrence of the genus in Pará State, Brazil.

## Material and methods

Morphological terminology follows that of Engel (2001, 2009) and Michener (2007), while the format for the description follows those of Engel et al. (1997) and Engel and Brooks (1999). The higher classification of Augochlorini is that of Engel (2000). Abbreviations used for common morphological terms are: S, metasomal sternum; T, metasomal tergum; F, flagellomere; DS, diameter of the antennal scape; and OD, ocellar diameter (based on the median ocellus). Measurements and proportions are adapted from Moure and Sakagami (1962): body length, head length and width, upper and lower interocular distances, and ocellocipital distance. Photomicrographs were prepared using a Nikon D1x digital camera attached to an Infinity K-2 long-distance microscope lens.

## Systematics

### Tribe Augochlorini Beebe, 1925. Subtribe Augochlorina Beebe, 1925. Genus Chlerogelloides Engel, Brooks, & Yanega, 1997

#### Chlerogelloides nexosa

Oliveira, Engel, & Mahlmann sp. n.

urn:lsid:zoobank.org:act:C5A48B27-0DC1-4AC6-9C17-9AD8E9D3C617

http://species-id.net/wiki/Chlerogelloides_nexosa

Figs 1 –3 5 7–10

##### Holotype.

♂, Brazil, Pará (Melgaço, Reserva Caxiuanã, Estação Ecológica Ferreira Penna, 01°43'S, 51°29'W, 18–21.ix.2011 [18–21 September 2011], Rech, Coelho, Correa & Carmo *Leg*. // coletada na flor: *Cordia nodosa* Lam. (Boraginales - Cordiaceae) – durante o curso de Polinização 2011 // Holotype male *Chlerogelloides nexosa* Oliveira, Engel & Mahlmann. The specimen is deposited in the Entomological Collection of the Museu Paraense Emílio Goeldi (MPEG), in Belém, Pará, Brazil.

##### Paratypes.

1♂, with same label data as holotype and deposited in the Entomological Collection of the Zoological Museum of the Federal University of Bahia (MZUFBA), in Salvador, Bahia, Brazil. 5♂♂, Guyane Française [French Guiana], Saül, Carbet ONF de Galbao, 27.viii.2003 [27 August 2003], on *Gonzalagunia dicocca*, leg. J. Munzinger; deposited in the Department of Entomology, Royal Belgian Institute of Natural Sciences, Brussels, Belgium and one in the Division of Entomology, University of Kansas Natural History Museum, Lawrence, Kansas, USA. 1♂, Guyane Fr. [French Guiana], Patawa, viii.1999 [August 1999], PM; deposited in the Department of Entomology, Royal Belgian Institute of Natural Sciences, Brussels, Belgium.

##### Diagnosis.

Integument predominantly honey yellow with metallic olive green highlights only on the head, mesoscutum, mesoscutellum, and metanotum (Figs 2, 3); postgena uniformly covered by plumose setae except; mesotrochanter without prominent ventral tubercle and not strongly bent (Fig. 5); mesofemur greatly expanded and flattened along inner surface, with a prominent tubercle on ventral surface in apical third (Fig. 5); mesotibia with inner surface twisted and flanked by non-contiguous, ill-defined carinas converging medioapically (Fig. 5); mesobasitarsus about twice as long as wide and weakly concave on inner surface; male terminalia as in figures 7–10.

##### Description.

♂: *Structure*: Total body length 6.60 mm; forewing length 4.50 mm. Head elongate, length 1.80 mm, width 1.47 mm (Figs 2, 3); clypeus longer than maximum width, length 0.60 mm, width 0.47 mm, almost entirety of clypeus (85%) set below lower tangent of compound eyes; frontal line weakly carinate between antennae, becoming a faintly impressed line from there to median ocellus; antennal scape relatively short (excluding basal bulb), length 0.50 mm; pedicel about as long as F1; F1 wider than long, slightly longer than F2; F2–F5 slightly wider than long; F6–11 progressively becoming longer than previous flagellomeres; distance from median ocellus to lateral ocellus 0.05 mm; between lateral ocelli 0.20 mm; ocellocular distance 0.22 mm (1.46× ocellar diameter); compound eye about 3.25x wider than gena in profile (width of compound eye 0.65 mm, width of gena 0.20 mm), beginning a little below middle of compound eyes. Intertegular distance 0.95 mm; mesoscutellum less than twice as long as metanotum (mesoscutellum length 0.35 mm, metanotum length 0.20 mm); dorsal surface of propodeum faintly concave, elongate (as for the genus: *vide* Engel et al. 1997; Engel and Brooks 1999; Engel 2000). Mesofemur greatly enlarged, approximately twice as long as wide (measurement made on internal surface: length 1.2 mm, width 0.55 mm), with flattened inner surface (Fig. 5), ventrally ridged along external border from apex basad to ventral tubercle in apical third; mesotibia slightly twisted on internal surface (in posterior view), with two non-contiguous carinae along borders and converging medioapically, one from base of mesotibia and with a small elevation near apex, other more apical on external border, both carinae terminating at a weakly depressed medioapical area from which spur articulates; mesobasitarsus twice as long as wide, flattened on dorsal surface and with carinae bordering weakly depressed inner surface. Forewing pterostigma very long, almost as long as first submarginal cell; hind wing with basal hamuli arranged 2-1, distal hamuli arranged 2-1-2. Male terminalia as in figures 7–10.

*Sculpturing*: Integument smooth and polished, faintly imbricate in some places, with sparse small to minute punctures, except face above level of antennal toruli with punctures more closely spaced as well as on mesoscutum, mesoscutellum, and metanotum; punctures separated by 2–5× a puncture diameter on mesepisternum; dorsal surface of propodeum smooth, polished, and glabrous.

*Coloration*: Integument predominantly honey yellow with brownish areas laterally pronotum near pronotal lobe, dorsal surface of propodeum, apex of metafemur, external surface of metatibia, apical third of T1, apical two-thirds of T2–T3, and T4–T7 with yellowish areas slightly darker than elsewhere (darker areas wider and longer in paratype). Head metallic olive green except honey yellow on clypeus (sometimes with some brown areas extending from base in paratypes from French Guiana), labrum, mandible, malar area, and antennal scape; pedicel flagellum dark brown. Mesoscutum, mesoscutellum, and metanotum metallic olive green, less shiny than on head; axilla dark brown with metallic reflections; tegula translucent brown; axillary sclerites brown, base of C+Sc honey yellow otherwise wing venation brown to dark brown; pleura honey yellow except mostly brown on hypoepimeral area, with weak metallic green highlights (almost imperceptible in most places); wing membranes hyaline, slightly and faintly infumate apically, with some iridescence depending on lighting.

*Pubescence*:Pubescence largely consisting of golden simple setae. Head with scattered, largely simple setae, those on supraclypeal area, vertex, gena, and postgena longer; setae dorso-apically on scape longer, between one-third and one-half DS, remainder much shorter; a few short, branched setae in lower paraocular area and surrounding upper border of supraclypeal area; gena with uniformly distributed branched setae, setae with branches arising from one side of rachis in apical two-thirds; setae along borders with compound eyes very short. Mesosomal setae generally simple except more plumose around pronotal lobe and surrounding propodeal spiracle; disc of mesoscutum with relatively short and sparse setae, shorter than those of mesepisternum, although denser than on the latter; disc of mesoscutellum with setae little longer than those of mesoscutum, distinctly longer along posterior margin; metanotum with yellowish, short, plumose setae intermixed with longer, branched, golden setae, laterally longer, about 1.5DS; lateral and posterior surfaces of propodeum with long (about 2DS), largely-simple, scattered setae, although less numerous than on mesoscutum. Leg pubescence typical for male Augochlorini except distal half of anterior surface of procoxa with dense, long, branched setae, such setae about 1.2DS; anterior area formed by carinae along ventral surface of mesobasitarsus glabrous and separate from posterior area covered by minute setae. Wing membranes uniformly pilose. Metasoma generally with sparsely-scattered, simple, long setae, on T1–T3 simple setae mostly distributed across discs, with apical margins glabrous, such setae varying from 1–1.5DS in length, setae becoming progressively longer on more apical segments, on T4–T7 with short, dense, plumose setae intermixed with long setae.

♀: Unknown.

**Figure 1.**
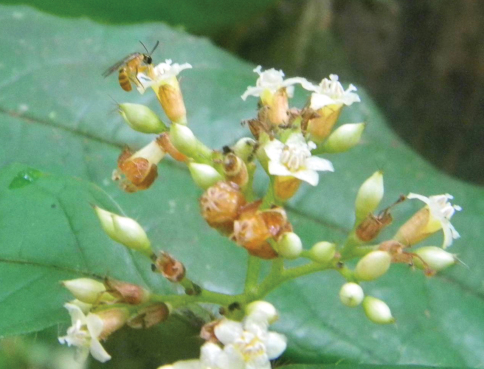
Male of *Chlerogelloides nexosa*sp. n.collecting resources at flowers of*Cordia nodosa* Lam. (Boraginaceae) at the Estação Ecológica Ferreira Penna (Caxiuanã Reserve, Melgaço, Pará, Brazil). Photograph by Rech et al. (in press).

**Figures 2–3.**
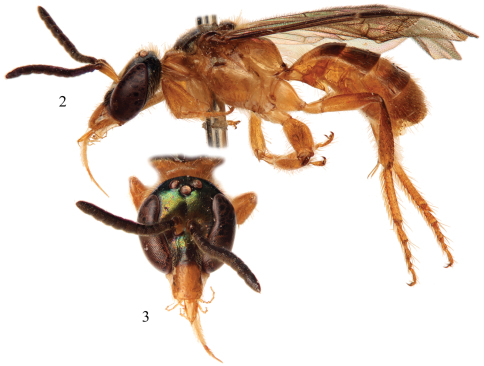
Photomicrographs of male paratype of *Chlerogelloides nexosa* sp. n. **2** Lateral habitus **3** Facial view.

**Figures 4–6.**
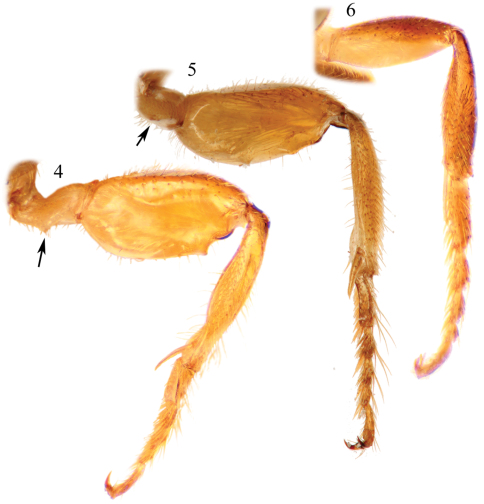
Male midlegs of species of *Chlerogelloides*. **4** *Chlerogelloides femoralis* Engel et al. **5** *Chlerogelloides nexosa* sp. n. **6** *Chlerogelloides simplex* Engel and Brooks.

**Figures 7–10.**
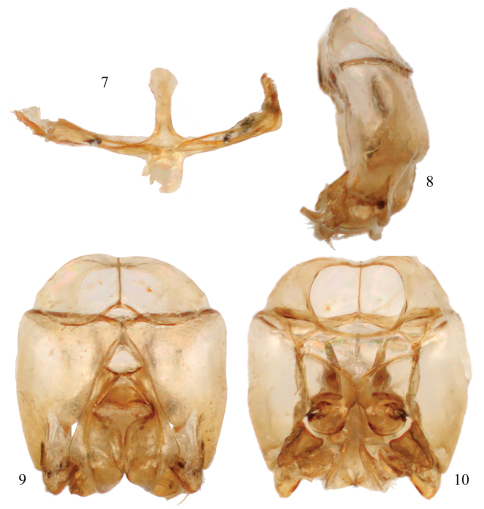
Male terminalia of *Chlerogelloides nexosa* sp. n. **7** Hidden and fused sterna VII+VIII **8** Genital capsule, lateral view **9** Genital capsule, dorsal view **10** Genital capsule, ventral view.

##### Etymology.

The specific epithet is taken from the Latin word *nexosus*, meaning “complicated”, and is a reference to the complex morphology of this and other species in the genus.

##### Comments.

A single female, collected in French Guiana with one of the males, is indistinguishable from that of *Chlerogelloides simplex* (specimen from Patawa, deposited in Brussels). It is possible that it is a female of *Chlerogelloides nexosa*, but given that definitive *Chlerogelloides simplex* is known from the same area we cannot be certain that it is a definitive female of the new species. Only further collecting in the region will be able to determine the correct association of females for this complex genus of bees.

### Key to species of Chlerogelloides

The present key is modified from that provided by Engel and Brooks (1999). Females are presently unknown for *Chlerogelloides nexosa* sp. n.

**Table:** 

1	Males	2
–	Females	4
2	Mesofemur greatly swollen and with one or two inner apical teeth (Figs 4, 5); metallic reflections of head and mesosoma tending toward green (Figs 2, 3, 13, 14); male terminalia as in figures 7–10 or 19–22	3
–	Mesofemur simple, unmodified (Fig. 6); metallic reflections of head and mesosoma tending toward blue (Figs 11, 12); gena lacking distinctive tuft of setae; face above level of antennae with minute punctures closely packed; apical quarter to one-third of clypeus yellow; axilla dark brown; male terminalia as in figures 15–18	*Chlerogelloides simplex* Engel & Brooks
3	Mesotrochanter with strong inner projection (Fig. 4); gena with distinctive tuft of long, plumose setae; face above level of antennae with minute punctures widely scattered over glabrous integument; apical two-thirds of clypeus yellow (Fig. 14); axilla yellow	*Chlerogelloides femoralis* Engel et al.
–	Mesotrochanter without strong inner projection (Fig. 5); gena without tuft of long, plumose setae; face above level of antennae with minute punctures closely packed; clypeus entirely but sometimes with extensive areas of brown extending from base (Fig. 3); axilla brownish	*Chlerogelloides nexosa* sp. n.
4	Clypeus and basal area of propodeum yellow	*Chlerogelloides femoralis* Engel et al.
–	Clypeus and basal area of propodeum brown	*Chlerogelloides simplex* Engel & Brooks

**Figures 11–12.**
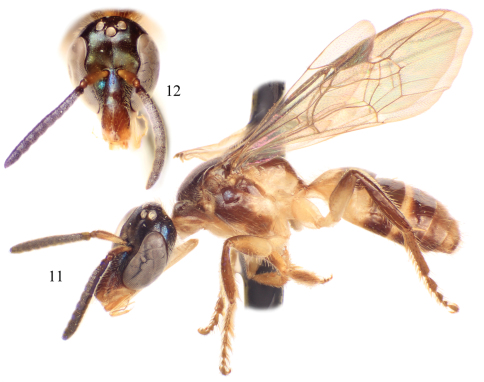
Male of *Chlerogelloides simplex* Engel and Brooks. **11** Lateral habitus **12** Facial view.

**Figures 13–14.**
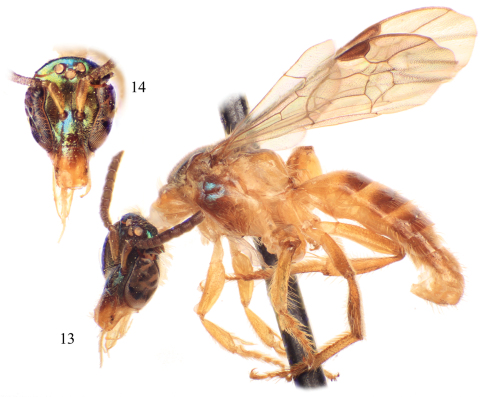
Male of *Chlerogelloides femoralis* Engel et al. **13** Lateral habitus **14** Facial view.

**Figures 15–22.**
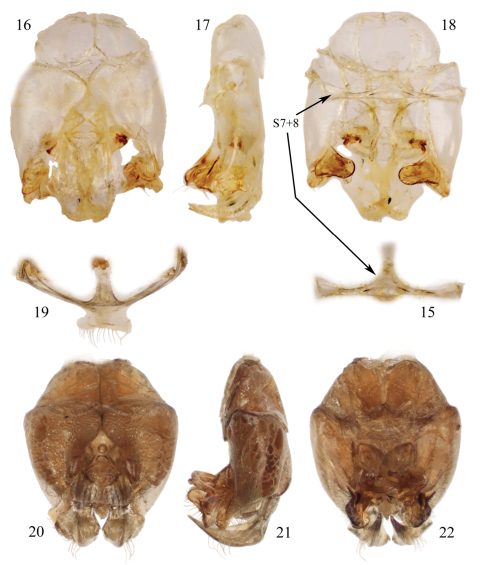
Male terminalia of species of *Chlerogelloides* (**15–18** *Chlerogelloides simplex* Engel and Brooks; **19–22** *Chlerogelloides femoralis* Engel et al.). **15** Hidden and fused metasomal sterna VII+VIII (in situ with capsule in figure **18**) **16** Genital capsule, dorsal view **17** Genital capsule, lateral view **18** Genital capsule, ventral view (with hidden sterna VII+VIII in situ). **19** Hidden and fused sterna VII+VIII **20** Genital capsule, lateral view **21** Genital capsule, lateral view **22** Genital capsule, ventral view.

## Discussion

The three known species of *Chlerogelloides* are all quite similar, immediately noticeable for their modified, elongate clypeus which is basally intruded upon by a deeply acute and projecting epistomal sulcus, such that the narrowed epistomal lobe nearly reaches the clypeal apex. In addition, the species share a short malar space, a pronotum in which the dorsal surface is expanded, and a serrate inner metatibial spur in males and females (Engel et al. 1997; Engel and Brooks 1999; Engel 2000). The bodies are largely yellow but with significantly regions of dark to brilliant metallic coloration, usually green but also blue and coppery (Figs 2, 3, 11–14). When the genus was first described, the sole species known (*Chlerogelloides femoralis*) had males with considerably modified midlegs, particularly the mesotrochanter, mesofemur, mesotibia, and mesobasitarsus (Fig. 4). Some of these modifications are now known to be shared with *Chlerogelloides nexosa* sp. n., such as the greatly enlarged (although somewhat compressed along the anterior-posterior axis) mesofemora with ventral tubercles or teeth and about twice as long as wide, the inner ridges and apically depressed surfaces of the mesotibiae, and the concave inner surfaces of the mesobasitarsi (Fig. 5). The mesotrochanter of *Chlerogelloides femoralis* is strongly bent and has a strong inner tubercle (Fig. 4), while the same podite in *Chlerogelloides nexosa* sp. n. lacks such a prominent modification, at most showing a weak swelling (Fig. 5). The mesobasitarsus of *Chlerogelloides femoralis* is about as long as wide and somewhat triangular, while it is longer than wide and more rectangular in *Chlerogelloides nexosa* sp. n. In contrast, the midlegs of *Chlerogelloides simplex* are like those of other Augochlorini (Fig. 6), particularly the normal mesofemora which are about four times as long as wide. In *Chlerogelloides nexosa* sp. n. and *Chlerogelloides femoralis* the metallic reflections of the head and mesosoma tend to be green while in *Chlerogelloides simplex*such areas are more bluish, although green highlights are indeed present in the few known specimens. Males of *Chlerogelloides nexosa* sp. n. have the gena uniformly covered by plumose setae in which the branches arise from the rachis on one side in the apical two-thirds, while males of *Chlerogelloides femoralis* have such setae clustered into a distinctive tuft midway along the gena. Other differences between *Chlerogelloides femoralis* and *Chlerogelloides nexosa* sp. n. include the smaller ocellocular distance and narrower gena (by comparison to the width of the compound eye) in the latter species. Lastly, the terminalia of *Chlerogelloides nexosa* sp. n. tend to more closely resemble those of *Chlerogelloides simplex* rather than *Chlerogelloides femoralis*. Detailed descriptions of *Chlerogelloides femoralis* and *Chlerogelloides simplex*were given relatively recently by Engel et al. (1997) and Engel and Brooks (1999), and that material is not repeated herein.

At present the genus is known only by a meager diversity and relative paucity of material, despite the seemingly wide range of the lineage across South America. Accordingly, it is premature to attempt too contemplative of an investigation into the evolution of this genus, particularly given that the resulting three-taxon statement would have little explanatory power in the absence of more complete life-history or biological data for the constituent species. Nonetheless, if we presume that the modified midlegs of *Chlerogelloides femoralis* and *Chlerogelloides nexosa* sp. n. represent a derived feature indicating a close relationship between these two species, then *Chlerogelloides simplex* would appear to fall basal within the genus. This assumption seems safe for the moment given that such modifications of the midleg podites are not known in related or other genera of Augochlorini (Engel 2000). It would be significant to discover the uses of these elaborate adaptations as used by the male during courtship and mating, and melittologists should be aware of these bees as they undertake observational studies in those regions where individuals are known to occur. Perhaps more interesting and feasible at this stage would be continued work on the relationship of the genus as a whole in relation to other lineages of Augochlorini. Engel and Brooks (1999) and Engel (2000) highlighted a putative relationship between *Chlerogelloides* and *Chlerogella* + *Ischnomelissa*. This hypothesis has not been supported by preliminary molecular work and *Chlerogelloides* does seem to occupy a rather isolated place among augochlorine genera, at least phenotypically. Intensive sampling is needed in order to increase the material available for the genus (for both molecular and morphological studies), more thoroughly document the distribution of its species, and to bring to light its presumably unique biology.

## Supplementary Material

XML Treatment for Chlerogelloides nexosa
